# Preserved Microarrays for Simultaneous Detection and Identification of Six Fungal Potato Pathogens with the Use of Real-Time PCR in Matrix Format

**DOI:** 10.3390/bios8040129

**Published:** 2018-12-13

**Authors:** Maksim Nikitin, Ksenia Deych, Inessa Grevtseva, Natalya Girsova, Maria Kuznetsova, Mikhail Pridannikov, Vitaly Dzhavakhiya, Natalia Statsyuk, Alexander Golikov

**Affiliations:** 1 GenBit LLC, Nauchny pr., 20, Bld. 4, Moscow 117246, Russia; nikitin@genbitgroup.com (M.N.); ksedeych@gmail.com (K.D.); grevtseva@genbitgroup.com (I.G.); golikov@genbitgroup.com (A.G.); 2 All-Russian Research Institute of Phytopathology, Institute Str., 5, Bolshie Vyazemy 143050, Russia; ngirsova@yandex.ru (N.G.); kuznetsova@vniif.ru (M.K.); mikhail.pridannikov@yahoo.com (M.P.); dzhavakhiya@yahoo.com (V.D.); 3 Centre of Parasitology, Severtsov Institute of Ecology and Evolution, Russian Academy of Sciences, Leninskii Prospect 33, Moscow 119071, Russia

**Keywords:** plant pathogen diagnostics, qPCR microarrays, potato, *Spongospora subterranea*, *Rhizoctonia solani*, *Alternaria solani*, *Alternaria alternata*, *Colletotrichum coccodes*, *Fusarium* spp.

## Abstract

Fungal diseases of plants are of great economic importance causing 70–80% of crop losses associated with microbial plant pathogens. Advanced on-site disease diagnostics is very important to maximize crop productivity. In this study, diagnostic systems have been developed for simultaneous detection and identification of six fungal pathogens using 48-well microarrays (micromatrices) for qPCR. All oligonucleotide sets were tested for their specificity using 59 strains of target and non-target species. Detection limit of the developed test systems varied from 0.6 to 43.5 pg of DNA depending on target species with reproducibility within 0.3−0.7% (standard deviation). Diagnostic efficiency of test systems with stabilized and freeze-dried PCR master-mixes did not significantly differ from that of freshly prepared microarrays, though detection limit increased. Validation of test systems on 30 field samples of potato plants showed perfect correspondence with the results of morphological identification of pathogens. Due to the simplicity of the analysis and the automated data interpretation, the developed microarrays have good potential for on-site use by technician-level personnel, as well as for high-throughput monitoring of fungal potato pathogens.

## 1. Introduction

Potato is the fourth main staple crop in the world with global production exceeding 375 million metric tons in 2016 [1]. The crop is grown in more than 100 countries and is crucial for welfare of billions of people, so its sustainable production is extremely important for global food security. However, potato suffers from a number of pests and diseases that cause significant yield reduction. Estimated actual potato yield losses caused by the above-mentioned biotic factors comprise 40% [2]. Among those 14% is caused by various microbial diseases [3].

Fungi and oomycetes form a significant part of economically important microbial pathogens; they may cause up to 70–80% of losses resulted from microbial diseases globally [2]. In the case of potato, most economically important diseases of such origin are: late blight caused by *Phytophthora infestans* (Mont.) de Bary (annual global yield losses of 16%, though may reach 70–100% in the case of insufficient protection [4]); stem canker and black scurf caused by *Rhizoctonia solani* Kühn (up to 30% of marketable yield losses [5]); powdery scab caused by *Spongospora subterranea* (Wallr.) Lagerh. f. sp. *subterranea* J. A. Toml. ($13.4 M of estimated annual losses in Australia alone [6]); wart disease caused by a quarantine pest *Synchytrium endobioticum* (up to 50–100% of yield losses on susceptible cultivars [7]); early blight/brown spot disease complex caused by *Alternaria solani* (Ellis and G. Martin) L.R. Jones and *A. alternata* (Fr.) Keissl. (up to 40% yield losses [8]); black dot caused by *Colletotrichum coccodes* (Wallr.) S.J. Hughes (up to 22–30% of yield losses [9]); dry rot of tubers caused by several *Fusarium* species (up to 25% storage losses [10]), etc.

Advanced disease diagnostics are very important to minimize disease-induced potato losses during production and storage, and to enhance crop productivity. Sensitive and accurate detection of potato pathogens provides for appropriate diseases’ control and prevents their outbreaks and spread; comprehensive diagnostics is also very important in case of latent or masked infections, or when visual discrimination between different diseases is complicated. Thus, visual discrimination between common scab (*Streptomyces* spp.) and much more dangerous powdery scab (*Spongospora subterranea*) can be difficult, since mature lesions of common scab are often confused with powdery scab manifestations [11]. In addition, correct diagnostics can provide proper plan of pesticide application, thus avoiding unnecessary treatments in case, when visual signs of infection may be confused with those from any abiotic factor; e.g., leaf lesions caused by early blight are very similar to the symptoms of ozone toxicity or deficiency in some microelements, such as manganese, zinc, and magnesium [12]. 

Traditional diagnostics of microbial pathogens is based on visual observation and characterization of symptoms followed by microscopic examination and culture-based morphological approaches. These methods are time-consuming, laborious, and require advanced skills in taxonomy and morphology of pathogens. Molecular-based direct detection techniques, such as immunoassay and polymerase chain reaction (PCR), permit more sensitive, accurate, and faster identification of pathogens. PCR-based technologies provide high sensitivity and specificity of pathogen identification and are most popular nowadays. They include conventional PCR, real-time PCR, nested PCR, reverse-transcription PCR, multiplex PCR, etc. [13]. Technologies based on monoclonal antibodies (ELISA and lateral flow devices (LFD)) are also widely used in plant pathogen diagnostics; e.g., the number of ELISA tests used yearly for this purpose worldwide reaches 10 M [14]. Some other technologies suitable for accurate identification of plant pathogens in stationary diagnostic laboratories have been developed, including immunofluorescent assay and DNA arrays [15,16,17,18].

Current climatic changes occurring in many regions affect plant resistance to pathogens, have an impact on the incidence and severity of potato diseases, and, along with globalization of international trade, provoke changes in their virulence and aggressiveness, as well as invasion of pathogens (including quarantine species) into new areas [19,20]. Under such conditions, early and rapid disease diagnostics becomes especially important as a tool to prevent or localize disease outbreaks and choose optimal and accurate control strategies. In the case of quarantine pests or the risk of epidemics, loss of time caused by necessity to send suspicious samples to stationary diagnostic laboratories and to receive answer in 2–3 days, or even 1–2 week (if the laboratory is located far from the field that is a common situation for developing countries), can be crucial and inadmissible. Thus, reliable and sensitive diagnostic tools suitable for use by unskilled personnel, field laboratories, or on-the-spot directly are of great demand. For end users, such diagnostic systems should fit the following criteria:

- cost of analysis should not be too high;
- analysis should be rapid, sensitive and specific;
- multiplex detection of a number of pathogens; and
- procedure of analysis should be simple and does not require special facilities and conditions;
- diagnostic kits should not fall under special storage and transportation limitations.

Most conventional methods for plant pathogens diagnostics do not completely meet these requirements. LFD use is rapid, very simple, and perfectly suited for on-the-spot application. However, like other immunodetection technologies, this method is less sensitive in relation to bacteria and fungi, has some cross-reactivity issues, and, until recently, did not provide multiplexity [21]. The cost of ELISA and IFA diagnostic kits is usually higher than for PCR-based technologies, while their shelf life is limited and requires special storage conditions [13]. PCR-based techniques require skilled personnel, are sensitive to cross-contamination and conditions of DNA isolation and amplification, and provides only limited multiplexing; in addition, PCR reagents also require special storage conditions, while equipment is often expensive and does not suit field laboratories [13,22]. DNA arrays provide excellent multiplexity but are used in stationary labs only.

Since all the above methods have drawbacks, a lot of efforts are made to improve these approaches or to develop alternative simple, reliable, and efficient field diagnostic techniques. Multiplex LFD [23], real-time PCR on portable amplifiers [24,25], simplification of DNA/RNA isolation technologies [26,27], LAMP PCR [28], various biosensors based on antibodies and DNA probes [29,30], and also microfluidic and stationary microchip PCR systems [31] are among the most promising.

In the case of potato, commercial kits currently available for disease diagnostics, directly on-the-spot or in field laboratories, are represented mainly by LFD and ELISA kits for detection of various potato viruses, some bacteria, and several fungi and oomycetes. There are also some FLASH PCR [32] kits suitable for detection of potato viruses and some bacteria and quarantine nematodes, as well as numerous real-time PCR kits for detection of a number of potato pathogens of different origin. In the case of fungi, the range of commercial diagnostic kits is quite limited and includes ELISA or LFD kits for detection of *P. infestans*, *R. solani*, and *S. subterranea*, as well as several PCR kits for detection of *S. endobioticum* and some *Fusarium* species. However, all these tools are based on the “one test—one pathogen” principle, i.e., they do not provide simultaneous diagnostics of multiple pathogens. Despite some reports on the development of multiplex LFD or PCR systems for the detection of a number of potato pathogens [23,33,34], these systems are not commercialized yet, i.e., they exist at the laboratory-scale only.

The given study presents a project started by the authors to provide an easy and convenient tool for multiplex field diagnostics of a wide range of potato pathogens based on the use of preserved disposable stationary qPCR microarrays with open microreactors. A special technology of immobilization and lyophilization of PCR mix components on the surface of reaction wells (provided by GenBit LLC, one of the partners in the project) and automatic analysis of results made it possible to significantly simplify the procedure of analysis: an operator should only prepare DNA/RNA extract and apply it into reaction wells of the multi-target matrix (sensor) under the sealing layer of mineral oil. In addition, this technology provides a long-term (up to six months) storage of preserved microarrays at room temperature without significant reduction of their analytic efficiency [35]. To date, test systems have been developed for the detection and identification of a range of potato pathogens, including eight viruses, six bacteria, four nematodes, and one oomycete [35,36,37]. The purpose of this study was the development of similar diagnostic systems for detection and identification of six economically important fungal pathogens of potato (*A. solani*, *A. alternata*, *R. solani*, *C. coccodes*, *S. subterranea*, and fungi belonging to the genus *Fusarium*).

## 2. Materials and Methods

### 2.1. Fungal Isolates, Cultivation, and DNA Extraction

Fungal isolates used in this study were provided by the State Collection of Plant Pathogenic Microorganisms, Indicator Plants and Differential Cultivars (SCPPM) of the All-Russian Research Institute of Phytopathology (Bolshie Vyazemy, Russia) and the “Microorganisms and Fungi” Division of the “Noah’s Ark” living systems depository of the Lomonosov Moscow State University (Moscow, Russia), and are listed in Table 1.

The ordered microorganisms were obtained from above-mentioned collections on agar slants. Petri plates with potato-dextrose agar were inoculated by pieces of mycelium and incubated for 5–10 days at room temperature in the dark. Genomic DNA was extracted from fungal mycelium using AmpliSens Ribo-Sorb DNA/RNA extraction kit (Russian Central Institute of Epidemiology, Moscow, Russia) in accordance with the manufacturer’s recommendations.

For extraction, a portion of mycelium with the upper layer of agar medium was collected from the area of ~1 cm^2^. DNA yield and purity were determined spectrophotometrically at 260 nm using a SmartSpec Plus spectrophotometer (BioRad, Berkeley, CA, USA). Obtained DNA samples were stored at −20 °C until use.

Since *S. subterranea* is an obligate intracellular parasite, isolation and maintenance of this pathogen in a pure culture is impossible [38]. Due to this fact, examination of the test system developed for detection of this pathogen, was carried out using samples of pathogen DNA isolated from two potato tubers (cvs. Red Scarlett and Colomba) infected with *S. subterranea* (Sss-RS and Sss-C, respectively). Tubers with visible signs of infection (pustules filled with powder-like brown spore mass) were revealed at the Leningrad regional branch of the Federal State Budgetary organization “Russian Agricultural Center” during analysis of a seed potato lot imported from Finland in 2017; analysis was carried out within the framework of a seed potato certification. Results of visual examination were then confirmed by microscopic examination of thin sections of infected tuber tissues for detection of spore balls. DNA isolation from samples with a confirmed presence of *S. subterranea* was performed as described above.

### 2.2. Oligonucleotide Design

To provide flexibility of microarray composition, the developed test systems should be efficient under the same “standard” amplification conditions and temperatures (see Section 2.4); this fact stipulated necessity to develop original primers and probes. PCR primers and fluorescent probes for each target pathogen were designed based on the internal transcribed spacer (ITS) region (*R. solani, S. subterranea*), *Alt_a1* gene (*A. solani, A. alternata*), *EF1* gene (*Fusarium* sp.), and *Tub2* gene (*C. coccodes*) using corresponding gene sequences from the NCBI database [39]. The sequences were aligned using a CLUSTAL 0(1.2.4) algorithm. Primer design and selection of a specific probe and reaction conditions were carried out using the Oligo 6 program [40]. Search of appropriate DNA region for primer and probe sequences was carried out using the following conditions: the length of resulting amplicons should be 70–300 bp, the preferable annealing temperature (T_a_) should be 60 °C, the difference in melting temperatures should not exceed 3 °C, and the number of mismatches between the amplified sequence and primers should not exceed 2. Additionally, possible dimer or hairpin formation was evaluated for each set of oligonucleotides. Theoretical examination of specificity of each primer pair was performed using a BLAST algorithm [41].

The resulted primers, as well as the probes for each primer pair labeled with 5′-fluorescent dye (FAM or ROX) and a 3′-quencher (BHQ-1 or BHQ-2), were manufactured by Biotech-Industria LLC. (Moscow, Russia). The length of resulted amplicons was evaluated with gel electrophoresis in 1% agarose with ethidium bromide staining.

### 2.3. Sample Preparation

For preliminary evaluation of the test system efficiency in a tube format, PCR master mix of total 20 μL consisted of 0.6 μL of dNTP mix (10 mM), 2 μL of the oligonucleotide mix (5 pmol/μL of each primer and 2.5 pmol/μL of a probe); 2.5 μL of a 10× Mg^2+^-containing PCR buffer, 0.9 μL of Taq DNA polymerase (5 U/μL, Sibenzyme Ltd., Novosibirsk, Russia), and 14 μL of DNase/RNase-free deionized water was prepared in 0.2-mL tubes. Five microliters of template DNA (~30–50 μg/mL) or deionized water (negative control) was added to PCR mastermix, and the resulted sample was analyzed using a DTLite 4 amplifier (DNK-Tekhnologiya, Moscow, Russia).

At the second stage of the study (sensitivity, specificity and reproducibility tests), PCR analysis was carried out using empty 48-well silicon microarrays (GenBit LLC, Moscow, Russia). After installation of a microarray into a holder cartridge, the whole reaction zone was accurately covered with a sealing layer of mineral oil (620 μL). For each test system, the above-mentioned PCR master mix was prepared in a volume corresponding to a number of required reaction wells. After addition of template DNA or deionized water (negative control), 1 μL of PCR mix was accurately loaded into each reaction well under the oil layer. A cartridge with the loaded microarray was inserted into an AriaDNA^®^ microchip amplifier (Lumex Marketing LLC, St.-Petersburg, Russia) for further real-time PCR.

**Figure 1:**
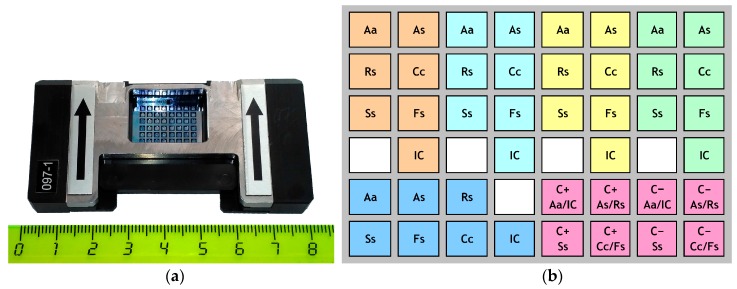
Microarray designed for diagnostics of fungal pathogens of potato: (**a**) 48-well microarray in a holder (the numbers on the ruler represent centimeters); (**b**) microarray topology for simultaneous analysis of five samples (shown by different colors). Indications: Aa, *Alternaria alternata*; As, *Alternaria solani*; Rs, *Rhizoctonia solani*; Cc, *Colletotrichum coccodes*; Ss, *Spongospora subterranea*; Fs, *Fusarium* spp.; IC, internal control sample; C+, positive control; C−, negative control.

At the third stage, the final testing was carried out using preserved 48-well silicon microarrays containing preliminary stabilized and freeze-dried components (master mixes, except for PCR buffer and target DNA) of the examined test systems (Figure 1). Preparation of the preserved microrrays was carried out by the GenBit LLC according to [42] with some modifications using a special technology developed independently of this study. Template DNA samples were mixed with 10× PCR buffer (Sibenzyme Ltd., Novosibirsk, Russia) at a 1:9 ratio; the resulting DNA concentration was ~1 μg/mL. After installation of a microarray into a holder cartridge and covering reaction zone with 620 μL of mineral oil, 1 μL of a sample DNA or deionized water (negative control) was added into each well under the sealing oil layer according to the particular matrix topology. Ready microarrays were inserted into an AriaDNA^®^ microarray amplifier for further real-time PCR.

### 2.4. Amplification Conditions and Data Analysis

The standard thermal cycling conditions for DNA amplification included initial denaturation step at 94 °C for 180 s followed by 45 cycles at 94 °C for 5 s and 60 °C for 30 s. The total amplification time was ~30 min. Data acquisition was automatically performed at the end of each cycle. The baseline was set in an automatic mode. Signal recording, calculation of critical threshold cycles (C_t_), and analysis of results were carried out automatically using an AriaDNA^®^ software package (Lumex-Marketing LLC, St.-Petersburg, Russia).

### 2.5. Validation of Developed qPCR Assays

The test systems were validated in several steps. First, for each target pathogen, the specificity of primer pairs was tested with 59 DNA samples of related and other target species, as well as with DNA samples of two different isolates of *Phytophthora infestans* (oomycete pathogen of potato, primer set for which has been developed earlier [35] and is planned to be added to the set of fungal pathogens of potato). All samples used were adjusted to final DNA concentration 1‒10 μg/mL; DNA of each species was tested in three repeats.

Subsequently, sensitivity of the developed test systems was determined using 5-6 of 10-fold serial dilutions of DNA extracted from pure cultures of the target species. DNA of each species was tested in three repeats for each concentration; all dilutions were arranged in the same microarray. According to the earlier report [35], we considered 35 to be the limit value of C_t_. With C_t_ above this value one cannot judge whether it is a false positive result or just a very low DNA presence.

Reproducibility of the approach was examined by analysis of the same sample of each target pathogen in 9 or 14 replications arranged on the same preserved microarray. DNA concentration used in this assay was adjusted to 1 μg/mL.

Finally, the assays were examined using 30 naturally infested field samples of potato and tomato. Leaves, stems, and tubers with visible manifestations of infection with target pathogens were collected from potato fields in several regions of Russia (see Section 3.6 for details). For each lesion one tissue sample was taken in the way so it included both infested and neighboring healthy tissues. DNA extraction from plant tissues was carried out as described in Section 2.1 for fungal mycelium; PCR analysis was performed as described in Section 2.4. Results of the analysis were compared with the results obtained by morphological identification of fungi [43].

## 3. Results

### 3.1. Primer Design

The best oligonucleotide set for each target species was selected (Table 2) upon alignment of DNA sequences (Figures S1–S3). The length of the resulted amplicons corresponded to the calculated values (data not shown).

Preliminary testing of the developed test systems in tube format showed their sufficient working capacity; all systems successfully detected target fungal species (data not shown). Each test system was then examined using the same sample DNA at four different annealing temperatures (T_a_) to evaluate its efficiency at the desired T_a_ (60 °C). According to results obtained (Table S1), T_a_ = 60 °C provided satisfactory results for all primer sets, i.e., the test systems were capable to provide good work efficiency under “standard” amplification conditions. 

### 3.2. Specificity Assay

All test systems were subjected to specificity examination at both development stage (using NCBI nucleotide-BLAST tool) and through laboratory testing. In the last case, each test system was checked for potential cross-reactions with genomic DNA of a range of the target and related species. For all species-specific primers sets, only DNA samples of target pathogen provided sufficient signal intensity, while signals from other species did not exceed threshold levels; in the case of a genus-specific Fus test system, all *Fusarium* species included in this study were successfully detected, while samples of other species did not show any amplification (Table 3, Figure S4). Thus, no cross-reactions with non-target species or false positive results were observed.

### 3.3. Sensitivity Assay and Regression Curves

**Figure 2:**
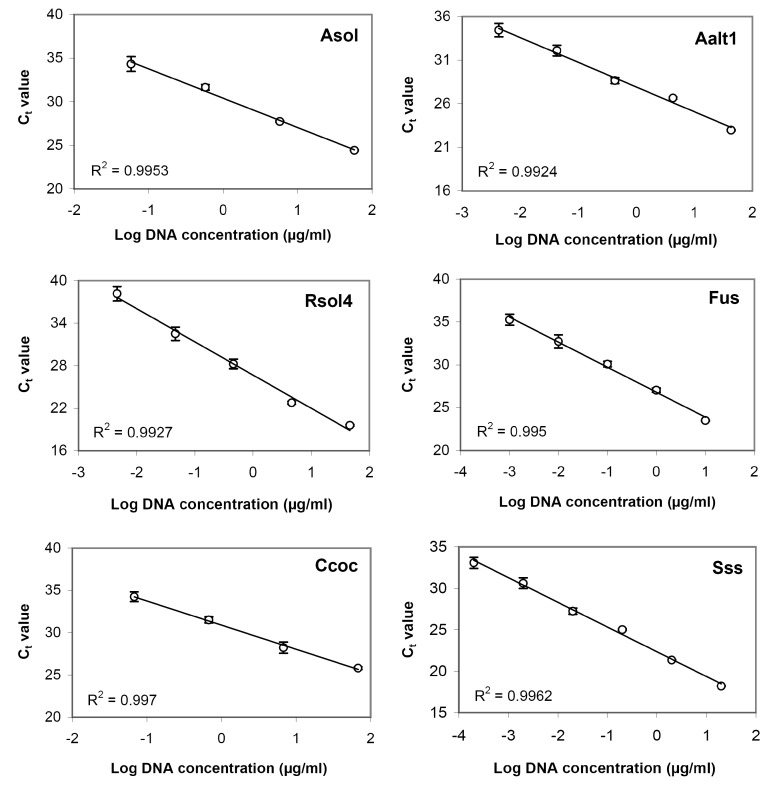
Standard regression curves of serial dilutions of DNA from the target potato pathogens. Test systems designations: **Asol4**, *Alternaria solani*; **Aalt1**, *A. alternata*; **Rsol4**, *Rhizoctonia solani*; **Ccoc**, *Colletotrichum coccodes*; **Sss**, *Spongospora subterranea*; **Fus**, *Fusarium* spp.

Examples of amplification curves obtained for serial dilutions of DNA of target potato pathogens are shown in Table 4 and Figure S5. Standard regression curves generated using serial DNA dilutions showed good linearity (Figure 2) with highly significant negative correlation between C_t_ values and DNA concentrations over the range used (R^2^ > 0.99 for all test systems), i.e., linear dynamic range of amplification was exhibited for the concentration ranges used. According the results of the performed assay, the lowest DNA concentrations providing C_t_ value below threshold value (C_t_ = 35), were about 43.5 (Asol4), 3.3 (Aalt1), 18 (Rsol4), 37 (Ccoc), 0.6 (Sss), and 1.7 (Fus) ng/mL. Since the reaction well volume of 48-well microarrays is 1 μL, these values corresponded to 43.5, 3.3, 18, 37, 0.6, and 1.7 pg of DNA.

### 3.4. Reproducibility Assay

**Figure 3:**
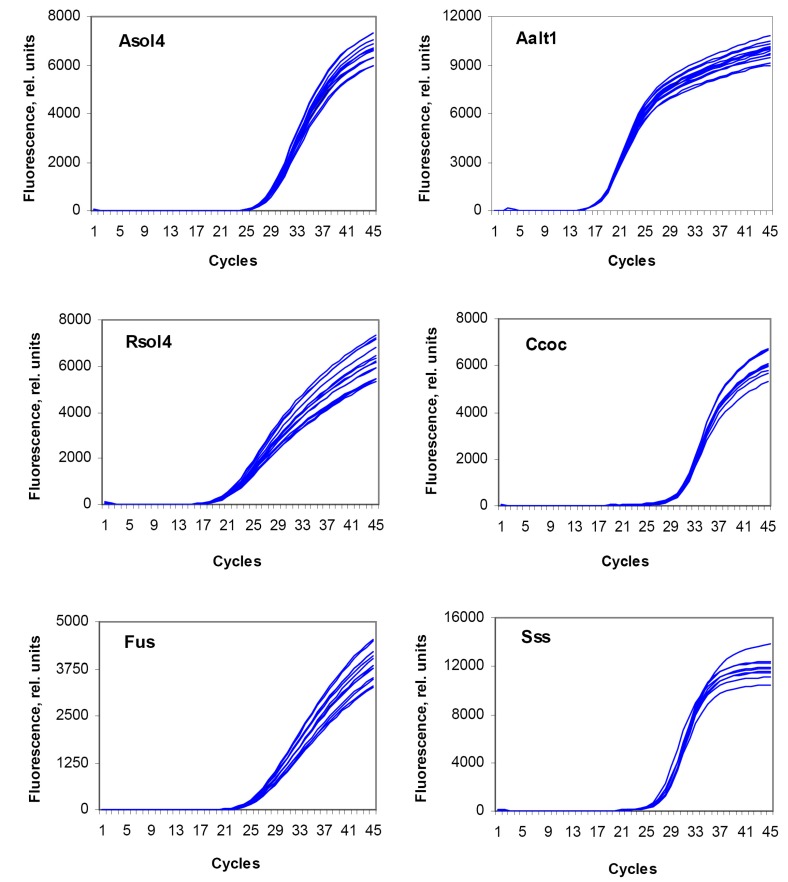
Reproducibility of the test systems for detection of the target potato pathogens. Test systems designations: **Asol4**, *Alternaria solani*; **Aalt1**, *A. alternata*; **Rsol4**, *Rhizoctonia solani*; **Ccoc**, *Colletotrichum coccodes*; **Sss**, *Spongospora subterranea*; **Fus**, *Fusarium* spp. Each species was analyzed in nine (Ccoc, Sss) or 14 (Aalt1, Asol4, Rsol4, Fus) repeats.

Results of the reproducibility assay for the developed test systems are shown in Figure 3. Mean C_t_ values are shown in Table 5. All test systems showed a good reproducibility; in all cases, the standard error for C_t_ values did not exceed 1%.

### 3.5. Comparison of the Working Efficiency of Fresh and Lyophilized Test Systems

Comparison of freshly prepared and lyophilized (ready-to-use) silicon microarrays for detection and identification of target potato pathogens was carried out upon completion of specificity, sensitivity, and reproducibility tests. After application of the required PCR components into reaction wells in accordance with the microarray topology (Figure 1b) and their lyophilization, detection efficiency of the resulted microarray was tested using DNA samples of target species; in parallel, the same samples were applied to a freshly prepared (non-lyophilized) microarray. C_t_ values and the round-off fluorescence level at the end of the analysis were used for a comparison. The experiment was carried out in two repeats.

**Figure 4:**
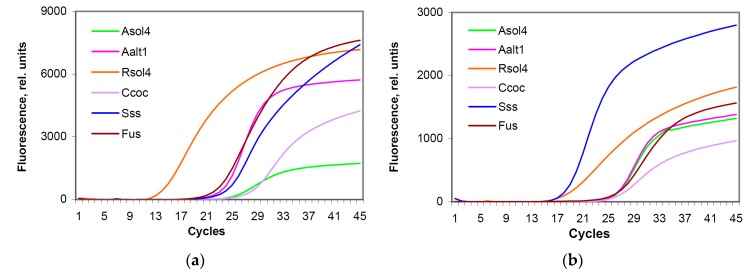
Parallel detection of target potato pathogens using (**a**) freshly prepared and (**b**) lyophilized test systems applied onto 48-well Si microarrays. Test systems designations: **Asol4**, *Alternaria solani*; **Aalt1**, *A. alternata*; **Rsol4**, *Rhizoctonia solani*; **Ccoc**, *Colletotrichum coccodes*; **Sss**, *Spongospora subterranea*; **Fus**, *Fusarium* spp.

Results are shown in Table 6 and Figure 4. For all test systems, C_t_ values obtained for both fresh and lyophilized microarrays were about the same, varying slightly, while final fluorescence signal of lyophilized test system dropped making 20–50% of the values obtained for fresh systems. Nevertheless, lyophilized microarrays provided a proper detection of pathogen DNA. Standard regression curves built on serial DNA dilutions showed good linearity for all test systems (Table 7, Figure S5) with highly significant negative correlation between C_t_ values and DNA concentration over the range used (R^2^ > 0.97). The lowest DNA concentration providing a C_t_ value below threshold value (C_t_ = 35), were 95 (Asol4), 5.8 (Aalt1), 22 (Rsol4), 84 (Ccoc), 2 (Sss), and 4 (Fus) ng/mL that corresponded to 95, 5.8, 22, 84, 2, and 4 pg of DNA. 

### 3.6. Validation of Test Systems with Field Samples

Results of examination of field samples with visible manifestations of target fungal pathogens are shown in Table 8. For majority of samples, results of the microscopic and PCR examination were similar. For T6 sample, microscopic examination revealed the presence of three pathogens, while PCR analysis on a microarray showed additionally the presence of *C. coccodes*. Analysis of L6 sample did not reveal *A. solani*, though microscopic examination confirmed the presence of single conidia of this pathogen. In both cases, the difference between the obtained results may be explained by a low limit presence of pathogen. Some samples (T4, T5, L7, S5, S13) did not show their actual presence of target pathogens under microscope and by PCR analysis, though having some manifestations of infection. That might be explained by the wrong initial diagnosis (presence of common scab instead of powdery scab in T4 and T5), or confusion with some abiotic factors or bacterial infection (L7, S5, S13).

In the most cases, C_t_ values obtained for target pathogens did not exceed 35, so were considered as positive results. Note that C_t_ values for the T6 and S11 (34.3 for *A. alternata* and 35.0 for *C. coccodes*, respectively) were very close or equal to the threshold value; though still being considered as being positive, they indicate a low infection load of the corresponding pathogens. In the case of L5, C_t_ = 36.1 for *A. alternata* that may be caused by either too low presence of the pathogen, or any nonspecific reaction. In such situation, no conclusion about the presence of a target pathogen can be made, and the result is considered to be negative. Since microscopic examination showed the presence of this fungus in the sample, we consider that the most possible reason of the obtained result is very low level of infection that is quite possible in the case of secondary colonization of necrotic lesions caused by *A. solani* with *A. alternata* [44].

## 4. Discussion

Being uncontrolled, fungal diseases of potato may have devastating effects on crop yield resulting in great economic losses. Like many soil-borne diseases, they can be symptomless during early infection stages and have long latent periods that complicate timely diagnostics. In addition, many fungal pathogens have similar symptoms and may be confused between themselves or with manifestations of various abiotic stresses. At the same time, early accurate detection and identification of fungal pathogens is the milestone of plant pathology, which provides an essential prerequisite to efficient implementation of disease management strategies. Another important moment are the benefits of early diagnostics performed on-site or in field laboratories, since it allows growers to make timely decisions concerning disease management strategies, to reduce impact of the disease, and, finally, save time and money.

In this study species-specific (genus-specific for *Fusarium* spp.) primers and probes were designed for detection of six important fungal pathogens of potato. The developed species-specific test systems were proved to be specific for the corresponding target species and did not show any cross-reactions with non-target fungi included into the study. At the same time, in the case of the test system for *S. subterranea* detection, some additional tests are desirable with some phylogenetically close plasmodiophorid species to confirm a high specificity of this assay. Since plasmodiophorids are obligate intracellular parasites, it is difficult to find them in the form of pure culture; nevertheless, we plan to do this study in the future.

The *Fusarium* genus-specific assay proved to be highly specific, detecting all of the reference *Fusarium* strains, with no cross-reaction with other fungal strains. The limitation of this assay is that the *Fusarium* genus includes both pathogenic and non-pathogenic species, i.e., a positive result may indicate the presence of either harmful, or harmless species. At the same time, while other target genera include mainly 1–2 species, which are pathogenic for potato, this genus includes at least 5–6 main species infecting potato (*F. sambucinum*, *F. oxusporum*, *F. avenaceum*, *F. solani*, *F. equiseti*, and *F. culmorum*) and also several less important species [45,46,47]. Therefore, use of species-specific assays for all *Fusarium* species pathogenic for potato may significantly reduce the number of samples, which can be tested per one microarray, and, therefore, increase the cost of analysis. We consider that the developed genus-specific test system can be used for primary on-site diagnostics, while the species-specific identification of *Fusarium* fungi (if they will be revealed) can be performed as the second stage. For this purpose, we plan to develop species-specific test systems for the main *Fusarium* pathogens, which then may be used not only for potato samples, but also for other vegetable crops and cereals, which significantly suffer from this fungal genus.

Standard regression curves demonstrated that the selected primer sets were highly accurate over the linear range of at least four orders of DNA content. In addition, all test systems showed good reproducibility (standard errors for C_t_ values did not exceed 0.7%).

Sensitivity is also an important characteristic of diagnostic test systems. In the case of *S. subterranea* and *A. alternata*, detection limits were 0.6 and 3.3 pg of DNA per reaction, respectively, that is close to the sensitivity of assays developed by other researchers [48,49], though is worse than some other published data (0.1 pg for both pathogens [50,51]). For *Fusarium* spp., a number of publications describe the development of diagnostic assays for single species, though only few describe genus-specific primer sets [52,53,54] or test systems detecting at least several *Fusarium* species causing dry rot of potato [55]. Sensitivity of the developed test system (1.7 pg of DNA) is inferior to that of some mentioned assays (0.2 [52] and 0.5 [55] pg of DNA or even one DNA copy per reaction [54]), though still remains at good diagnostic level. The other three test systems showed higher detection limits: 18, 37, and 43,5 pg of DNA for *R. solani*, *C. coccodes*, and *A. solani*, respectively. These values are higher than those for laboratory real-time assays developed by other authors (0.1‒1 pg for *A. solani* [51,56], 2‒20 pg for *C. coccodes* [57,58], and 0.1–2 pg for *R. solani* [59,60,61]), but still provide an adequate level of detection of target pathogens. Note that in our case, a primer selection process had several limiting conditions, the most important of which were the amplicon size and the efficient work of the developed assay at T_a_ = 60 °C. These conditions limited the range of appropriate variants, so in some cases we were not able to achieve high sensitivity of chosen primers; nevertheless, they still have quite good efficiency for the on-site testing of infected plants.

Parallel testing of the same DNA samples of target pathogens on freshly prepared and freeze-dried microarrays demonstrated that immobilization and freeze-drying of PCR mix components in reaction wells reduced the maximum fluorescence levels 2.5−3-fold (Table 7), which was expected; this effect might be caused by the immobilized state of freeze-dried DNA polymerase and oligonucleotides resulting in their conformational changes; in addition, the quenching effect may also appear due to a crowding of fluorescently labeled probe molecules. Nevertheless, it did not significantly affect C_t_ values, i.e., the procedure of preparation of preserved microarrays did not provide a significant negative effect on the working efficiency of the test systems. The further sensitivity test performed on the preserved microarrays showed the detection limits within the range of 2−6 pg of DNA for *S. subterranea*, *A. alternata*, and *Fusarium* spp. and 22−95 pg of DNA for *R. solani*, *C. coccodes*, and *A. solani* that was higher than in freshly prepared microarrays, but is still enough for a satisfactory detection level. Diagnostic efficiency of microarrays was successfully validated using infected field samples of potato plants and a parallel morphological identification of pathogens.

Thus, in this study we have successfully developed and validated test systems for diagnostics of six economically important fungal pathogens of potato intended for the use in preserved disposable real-time PCR microarrays. Such diagnostic microarrays provide simultaneous analysis of five samples for the presence of six pathogens. Due to small reaction volume (1 μL) and special technology for stabilization and freeze-drying of PCR mix components, the number of required procedures is significantly reduced, and the total time of analysis (including DNA extraction) takes only ~1.5 h. In addition, the use of preserved microarrays and automated data interpretation significantly simplifies the procedure and does not require highly-qualified personnel. This fact, together with a long shelf life of preserved microarrays at room temperature, provides an excellent suitability of microarrays for use by unskilled persons under conditions of field laboratories.

Despite obvious advantages of the proposed approach, there are also some issues that should be addressed in future. First, since all test systems arranged on the same microarray should work under the same amplification conditions, we developed original primers with similar optimal working temperatures and regimes. Due to this limitation, some of the developed primers showed increased detection limits. Though they are still suitable for the use in situation, when a potato grower finds some lesions on potato plants or tubers and wants to determine if there is a disease manifestation, which requires some control measures, their use for diagnostics of hidden diseases requires better sensitivity, so their further improvement should be done. Second, more fungal strains of target and non-target species should be tested to ensure specificity. Third, validation of the approach should be continued for a large number of infested field samples.

Among other possible directions of the further work, one should mention the development of test systems for detection of other fungal pathogens of potato, such as *S. endobioticum*, *Phoma* spp., *Pythium* spp. with their further combining with the earlier developed ones in the same microarrays. In addition, many of fungal pathogens of potato belong to soil-borne pathogens. They may develop special structures, such as spores and melanized hyphae to survive in soil for many years, so the proper detection of their presence in soil (especially quarantine species) is also very important for timely decisions regarding early-stage treatments or pre-plant assessments of the fields. Therefore, there is a need to develop an efficient protocol for detection of target pathogens in soil samples.

Due to simplicity of the approach we have developed, it can be used for pathogen detection by potato growers themselves without need to send samples to a diagnostic laboratory that could be quite away from the source. Flexibility of the microarray matrix topology and standard amplification conditions for all primer sets provide the possibility to combine test systems for various pathogens (fungi, bacteria, nematodes) depending on epidemiological situation at the point of use and on the needs of the customer (diagnostics of storage diseases, soil-borne pathogens, leaf infections, etc.). The multiplex mode of detection makes it possible to reveal combined infections and to perform high-throughput monitoring of potato infections on the basis of regional plant protection services. Due to above-mentioned reasons, the developed approach has a great potential for on-site use and will contribute to current diagnostic pipeline, as well as monitoring of potato pathogens.

## Figures and Tables

**Table 1:** Fungal isolates used in this study.

Species	Isolate Code	Year and Region of Collection	Host Plant	Source ^1^
*Alternaria solani*	100053	2009, Mariy El Republic, Russia	Potato	SCPPM
*A. solani*	043-021	– ^2^	–	MF
*A. solani*	044-051	–	–	MF
*A. alternata*	4l/6	2011, Republic of Mordovia, Russia	Barley	SCPPM
*A. alternata*	4k/2	2011, Republic of Mordovia, Russia	Barley	SCPPM
*A. alternata*	AK2	–	–	MF
*A. alternata*	HKK1	–	–	MF
*A. alternata*	12RKL9	2007, Ryazan region, Russia	Potato	MF
*A. alternata*	12RKL10	2007, Ryazan region, Russia	Potato	MF
*A. infectoria*	K100088	2009, Krasnodar region, Russia	Bindweed	SCPPM
*A. radicina*	K100011	2009, Moscow region, Russia	Carrot	SCPPM
*A. longipes*	K100055	2000, Moscow region, Russia	Tomato	SCPPM
*Rhizoctonia solani*	100006	2005, Moscow region, Russia	Potato	SCPPM
*R. solani*	100063	2011, Moscow region, Russia	Potato	SCPPM
*R. solani*	100106	2013, Leningrad region, Russia	Potato	SCPPM
*R. solani*	R15BKK nev25	2015, Vladimir region, Russia	Potato	SCPPM
*R. solani*	14BM rs2	2014, Vladimir region, Russia	Potato	SCPPM
*R. solani*	14KC man1	2014, Kostroma region, Russia	Potato	SCPPM
*R. cerealis*	1000025	2009, Ryazan region, Russia	Spring wheat	SCPPM
*Colletotrichum coccodes*	100004	2009, Moscow region, Russia	Potato	SCPPM
*C. coccodes*	100123(3)	2012, Voronezh region, Russia	Potato	SCPPM
*C. coccodes*	100124(3)	2013, Tula region, Russia	Potato	SCPPM
*C. coccodes*	100119(3)	2015, Orenburg region, Russia	Potato	SCPPM
*C. coccodes*	100120(3)	2015, Moscow region, Russia	Potato	SCPPM
*C. coffeanum*	100003	2008, Moscow region, Russia	Ornamental plants	SCPPM
*C. gloeosporioides*	100002	2009, Bryansk region, Russia	Blue lupine	SCPPM
*C. gloeosporioides*	100094	2013, Moscow region, Russia	Tatarian honeysuckle	SCPPM
*C. lilii*	100001	2009, Moscow region, Russia	Lily	SCPPM
*C. dematium*	100158	2017, Republic of Mordovia, Russia	Barley	SCPPM
*Fusarium avenaceum*	MOK-16-3	2016, Moscow region, Russia	Potato	SCPPM
*F. avenaceum*	110501	2003, Moscow region, Russia	Barley	SCPPM
*F. avenaceum*	U-08-8-1	2008, Ulyanovsk region, Russia	Wheat	SCPPM
*F. culmorum*	MKRS-15-3	2015, Moscow region, Russia	Potato	SCPPM
*F. culmorum*	100135(6-1)	2009, Moscow region, Russia	Potato	SCPPM
*F. culmorum*	100136(6-2)	2009, Moscow region, Russia	Potato	SCPPM
*F. gibbosum*	100130(7-2)	2014, Moscow region, Russia	Potato	SCPPM
*F. gibbosum*	100131(7-2)	2014, Moscow region, Russia	Potato	SCPPM
*F. gibbosum*	100132(7)	2009, Voronezh region, Russia	Potato	SCPPM
*F. heterosporum*	MOK-16-1	2016, Moscow region, Russia	Potato	SCPPM
*F. heterosporum*	100133(8)	2009, Moscow region, Russia	Potato	SCPPM
*F. oxysporum*	FO-1	1995, Moscow region, Russia	Potato	SCPPM
*F. oxysporum*	100139(9)	2014, Moscow region, Russia	Potato	SCPPM
*F. oxysporum*	100140(9)	2013, Bryansk region, Russia	Potato	SCPPM
*F. oxysporum*	RAM-14	2014, Moscow region, Russia	Potato	SCPPM
*F. sambucinum*	P-2-02	2002, Ryazan region, Russia	Barley	SCPPM
*F. sambucinum*	KRT 11-1 kch	2012, Krasnodar region, Russia	Wheat	SCPPM
*F. sambucinum*	100134(10)	2009, Lipetsk region, Russia	Potato	SCPPM
*F. solani*	FSL-9	2002, Moscow region, Russia	Potato	SCPPM
*F. solani*	100021	2001, Moscow region, Russia	Potato	SCPPM
*F. solani*	100137(11-1)	2009, Moscow region, Russia	Potato	SCPPM
*F. solani*	100138(11-2)	2009, Moscow region, Russia	Potato	SCPPM
*F. sporotrichioides*	KRT12-1kch	2012, Krasnodar region, Russia	Wheat	SCPPM
*F. sporotrichioides*	100141(12)	2009, Moscow region, Russia	Potato	SCPPM
*F. javanicum*	MKRS-15-1	2015, Moscow region, Russia	Potato	SCPPM
*F. sacchari*	RAM-16	2016, Moscow region, Russia	Potato	SCPPM
*Phytophthora infestans*	MVK 118a-07	2007, Moscow region, Russia	Potato	SCPPM
*P. infestans*	ATP-3.08	2008, Astrakhan region, Russia	Tomato	SCPPM

^1^ SCPPM: State Collection of Plant Pathogenic Microorganisms of the All-Russian Research Institute of Phytopathology, Bolshie Vyazemy, Russia; MF: “Microorganisms and Fungi” Division of the “Noah’s Ark” living systems depository of the Lomonosov Moscow State University, Moscow, Russia. ^2^ Not available.

**Table 2:** Species-specific primers and probes designed for the qPCR-based diagnostics of six fungal pathogens of potato.

Test System	Primers and Probe Sequences (5′–3′)	Length, Bases	T_m_ *, °C	Amplicon Size, bp
**Asol4** *Alternaria solani*	F: GGTCAGCGACGAGTAAGTT	19	59.4	71
	R: CAGATATACTAACGCTTTTCCA	22	60	
	Probe: ROX-CACGCTTTTCACCACCTTTTAC-BHQ2	22	66.7	
**Aalt1** *Alternaria alternata*	F: AGGAACCCTCGACTTCACCT	20	62.0	75
	R: TTCTCGCCACAGGAGTACCA	20	62.0	
	Probe: FAM- CTCTGCTCAGGCCGATAAGCT-BHQ1	21	66.0	
**Rsol4** *Rhizoctonia solani*	F: TTCACACCTGCTCCTCTTT	19	59.5	128
	R: TTCATCTGCATTTACCTTGG	20	60.8	
	Probe: FAM-TGCTTGGTTCCACTCAGCG-BHQ1	19	67.2	
**Ccoc** *Colletotrichum coccodes*	F: ACTTGTTCGAATAGGGTAACC	21	60.5	115
	R: TAGGGCACAGTCAGTAATTCA	21	60.5	
	Probe: FAM-AACCAGACAGACGCCAACGA-BHQ1	20	68.5	
**Sss** *Spongospora subterranea*	F: GCCTCTTTGAGTGTCGGTT	19	62	124
	R: AATCAGAAGCCAGAGACGC	19	62	
	Probe: FAM-TGTGCGTGGAAGGGGACTA-BHQ1	19	66	
**Fus** *Fusarium* spp.	F: TTGATCTACCAGTGCGGTG	19	60	369
	R: GATACCACGCTCACGCTC	18	60	
	Probe: FAM-TGAGCTTGTCAAGAACCCAGG-BHQ1	21	67	

* Theoretical melting temperature.

**Table 3:** Specificity test for individual DNA-based systems for diagnostics of fungal pathogens of potato.

Species	Isolate	C_t_ Values (C_t_ ± SE) Obtained for the Pathogens Included into the Specificity Test ^1^					
		Asol4	Aalt1	Rsol4	Fus	Ccoc	Sss
*Alternaria solani*	100053	27.23 ± 0.15	–	–	–	–	–
*A. solani*	043-021	24.79 ± 0.16	–	–	–	–	–
*A. solani*	044-051	25.23 ± 0.05	–				
*Alternaria alternata*	4l/6	–	20.62 ± 0.47	–	–	–	–
*A. alternata*	4k/2	–	21.13 ± 0.90				
*A. alternata*	AK2	–	21.52 ± 0.71				
*A. alternata*	HKK1	–	22.02 ± 0.96				
*A. alternata*	12RKL9	–	24.86 ± 0.01	–	–	–	–
*A. alternata*	12RKL10	–	22.93 ± 0.95				
*A. infectoria*	K100088	–	–	–	–	–	–
*A. longipes*	K100055	–	–	–	–	–	–
*Rhizoctonia solani*	100006	–	–	16.53 ± 0.32	–	–	–
*R. solani*	100063	–	–	12.61 ± 0.08	–	–	–
*R. solani*	100106			16.29 ± 1.73			
*R. solani*	R15BKK nev25			17.37 ± 0.24			
*R. solani*	14BM rs2			17.54 ± 0.46			
*R. solani*	14KC man1			20.29 ± 0.48			
*R. cerealis*	1000025			–			
*Colletotrichum coccodes*	100004	–	–	–	–	27.85 ± 0.37	–
*C. coccodes*	100123(3)					28.14 ± 0.15	
*C. coccodes*	100124(3)	–	–	–	–	25.24 ± 0.12	–
*C. coccodes*	100119(3)					25.94 ± 0.39	
*C. coccodes*	100120(3)					24.25 ± 0.65	
*C. coffeanum*	100003	–	–	–	–	–	–
*C. gloeosporioides*	100002	–	–	–	–	–	–
*C. gloeosporioides*	100094					–	
*C. lilii*	100001					–	
*C. dematium*	100158					–	
*Fusarium avenaceum*	MOK-16-3	–	–	–	25.13 ± 0.10	–	–
*F. avenaceum*	110501				32.32 ± 0.29		
*F. avenaceum*	U-08-8-1				23.86 ± 1.02		
*F. culmorum*	MKRS-15-3				31.60 ± 0.59		
*F. culmorum*	100135(6-1)				23.30 ± 0.12		
*F. culmorum*	100136(6-2)	–	–	–	30.68 ± 1.09	–	–
*F. gibbosum*	100130(7-2)	–	–	–	32.87 ± 2.13	–	–
*F. gibbosum*	100131(7-2)				26.11 ± 0.78		
*F. gibbosum*	100132(7)				26.44 ± 0.74		
*F. heterosporum*	MOK-16-1				31.06 ± 0.89		
*F. heterosporum*	100133(8)				32.70 ± 1.56		
*F. oxysporum*	FO-1				31.72 ± 0.29		
*F. oxysporum*	100139(9)				26.04 ± 0.45		
*F. oxysporum*	100140(9)	–	–	–	25.09 ± 0.52		
*F. oxysporum*	RAM-14				34.02 ± 0.59		
*F. sambucinum*	P-2-02				28.05 ± 0.16		
*F. sambucinum*	KRT 11-1 kch	–	–	–	24.04 ± 0.08	–	–
*F. sambucinum*	100134(10)				26.05 ± 0.49		
*F. solani*	FSL-9				33.39 ± 0.78		
*F. solani*	100021	–	–	–	33.72 ± 0.69	–	–
*F. solani*	100137(11-1)				32.40 ± 0.52		
*F. solani*	100138(11-2)				30.62 ± 1.29		
*F. sporotrichioides*	KRT12-1 kch				31.96 ± 1.25		
*F. sporotrichioides*	100141(12)				32.78 ± 0.96		
*F. javanicum*	MKRS-15-1				33.35 ± 0.84		
*F. sacchari*	RAM-16				33.65 ± 0.88		
*Phytophthora infestans*	MVK 118a-07	–	–	–	–	–	–
*P. infestans*	ATP-3.08	–	–	–	–	–	–
*Spongospora subterranea*	Field sample, Sss-RS	–	–	–	–	–	21.36 ± 0.05
*S. subterranea*	Field sample, Sss-C	–	–	–	–	–	20.53 ± 0.08

^1^ Blank cells indicate strains not included into the assay. “–” indicates zero amplification.

**Table 4:** Performance of the diagnostic systems for serial dilutions of DNA of target species.

Target Species	DNA Concentration, μg/mL	Threshold Cycle (C_t_) ^1^	Target Species	DNA Concentration, μg/mL	Threshold Cycle (C_t_)
*Alternaria solani*	58	24.42 ± 0.06	*Colletotrichum coccodes*	68	25.82 ± 0.15
	5.8	27.73 ± 0.08		6.8	28.23 ± 0.67
	0.58	31.64 ± 0.32		0.68	31.50 ± 0.35
	0.058	34.32 ± 0.84		0.068	34.24 ± 0.57
*Alternaria alternata*	43	22.97 ± 0.08	*Rhizoctonia solani*	46	19.59 ± 0.12
	4.3	26.66 ± 0.12		4.6	22.77 ± 0.20
	0.43	28.65 ± 0.34		0.46	28.25 ± 0.66
	0.043	32.09 ± 0.62		0.046	32.51 ± 0.94
	0.0043	34.43 ± 0.76		0.0046	38.16 ± 1.04
*Fusarium* sp.	10	23.5 ± 0.07	*Spongospora subterranea*	20	18.22 ± 0.05
	1	27.04 ± 0.24		2	21.36 ± 0.05
	0.1	30.08 ± 0.33		0.2	25.02 ± 0.10
	0.01	32.72 ± 0.78		0.02	27.25 ± 0.37
	0.001	35.26 ±0.64		0.002	30.62 ± 0.66
				0.0002	33.07 ± 0.64

^1^ Data are shown in the form of M ± SE, where M is a mean value and SE is the standard error calculated for three repeats.

**Table 5:** Mean C_t_ values obtained for the developed test systems for diagnostics of fungal pathogens of potato (reproducibility assay).

Test System	Number of Repeats	C_t_ Value (M ± SE)	SE (%)
**Asol4** (*Alternaria solani*)	14	27.23 ± 0.10	0.4
**Aalt1** (*Alternaria alternata*)	14	16.20 ± 0.06	0.3
**Rsol4** (*Rhizoctonia solani*)	14	20.24 ± 0.08	0.4
**Ccoc** (*Colletotrichum coccodes*)	9	27.39 ± 0.19	0.7
**Fus** (*Fusarium* spp.)	14	22.66 ± 0.09	0.4
**Sss** (*Spongospora subterranea*)	9	23.32 ± 0.17	0.7

**Table 6:** Comparison of working efficiency of freshly prepared and lyophilized microarrays for detection and identification of fungal pathogens of potato.

Test	Freshly Prepared		Lyophilized	
System ^1^	C_t_ (M ± SE)	Fluorescence Level at the End of Analysis	C_t_ (M ± SE)	Fluorescence Level at the End of Analysis
Asol4	24.06 ± 0.42	3000	26.50 ± 2.25	1300
Aalt1	25.70 ± 0.05	5700	24.56 ± 0.42	1400
Rsol4	19.54 ± 0.38	5600	18.80 ± 0.25	1900
Ccoc	27.25 ± 0.39	3500	26.61 ± 1.02	980
Fus	25.40 ± 0.25	7500	24.49 ± 0.88	1500
Sss	26.79 ±0.03	6800	26.56 ± 0.09	2200

^1^ Test systems designations: **Asol4**, *Alternaria solani*; **Aalt1**, *A. alternata*; **Rsol4**, *Rhizoctonia solani*; **Ccoc**, *Colletotrichum coccodes*; **Sss**, *Spongospora subterranea*; **Fus**, *Fusarium* spp.

**Table 7:** Performance of lyophilized diagnostic systems for serial dilutions of DNA of target species.

Target Species	DNA Concentration, μg/mL	Threshold Cycle (Ct)^1^	Target Species	DNA Concentration, μg/mL	Threshold Cycle (Ct)
*Alternaria solani*	136.4	26.50 ± 0.24	*Alternaria alternata*	43.5	20.64 ± 0.39
	13.64	29.23 ± 0.22		4.35	23.74 ± 0.32
	1.364	32.87 ± 0.41		0.435	27.73 ± 0.38
	0.1364	34.08 ± 0.86		0.0435	31.85 ± 0.59
	0.01364	37.18 ± 0.96		0.00435	35.51 ± 0.86
*Rhizoctonia solani*	18.32	18.40 ± 0.40	*Colletotrichum coccodes*	19.3	24.47 ± 0.38
	1.832	24.41 ± 0.70		1.93	29.84 ± 0.47
	0.1832	31.57 ± 0.98		0.193	34.09 ±0.55
	0.01832	34.33 ± 1.14		0.0193	37.10 ± 0.85
*Fusarium* sp.	44.6	22.42 ± 0.24	*Spongospora subterranea*	23.2	23.35 ± 0.35
	4.46	24.50 ± 0.33		2.32	26.46 ± 0.27
	0.446	28.54 ± 0.48		0.232	29.43 ± 0.39
	0.0446	31.73 ± 0.64		0.0232	32.73 ± 0.62
	0.00446	34.73 ± 0.62		0.00232	35.37 ± 0.76

^1^ Data are shown in the form of M ± SE, where M is a mean value and SE is the standard error calculated for three replications.

**Table 8:** Validation of a diagnostic efficiency of a qPCR microarray for detection of fungal pathogens of potato using field samples of potato leaves and tubers.

Sample No. ^1^	Origin ^2^	Visual Symptoms	Microscopic Examination ^3^	Real-Time PCR Analysis ^4^
T1	Leningrad region, Russia	Dry and dark surface lesions	Aalt Sss	Aalt (25.2) Sss (30.4)
T2	Leningrad region, Russia	Dry and dark surface lesions	Sss	Sss (31.5)
T3	Moscow region, Russia (Red Scarlett)	Black scurf (*Rhizoctonia solani*)	Rsol	Rsol (25.6)
T4	Tambov region, Russia	Superficial scabs (powdery scab suspected)	No spore balls of *S. subterranea*	–
T5	Omsk region, Russia	Superficial scabs (powdery or common scab suspected)	No spore balls of *S. subterranea*	–
T6	Unknown (bought in a supermarket)	Black scurf, superficial scabs and dry lesions	Sss Rsol Aalt (single conidia)	Sss (26.7) Ccoc (29.2) Rsol (21.4) Aalt (34.3)
T7	Leningrad region (Red Scarlett)	Black scurf, superficial dry lesions	Rsol Aalt Ccoc	Ccoc (31.4) Aalt (27.3) Rsol (20.4)
T8	Moscow region (Red Scarlett)	Dry rot	Fus (*F. oxysporum*)	Fus (17.6)
T9	Moscow region (Red Scarlett)	Dry rot	Fus (*F. oxysporum*)	Fus (21.4)
L1	Republic of Tatarstan, Russia	Brown spot disease	Aalt	Aalt (29.4)
L2	Republic of Kabardino-Balkaria, Russia ^5^	Brown spot disease	Aalt	Aalt (33.7)
L3	Moscow region, Russia (Red Scarlett)	Brown spot disease	Aalt	Aalt (27.7)
L4	Moscow region, Russia (Alpha)	Early blight/brown spot	Aalt	Aalt (34.1)
L5	Moscow region, Russia (Udacha)	Early blight/brown spot	Asol Aalt (single conidia)	Asol (26.8) Aalt (36.1)
L6	Tambov region (Alouette)	Early blight/brown spot	Asol (single conidia) Aalt	Aalt (28.3)
L7	Tambov region (Alouette)	Early blight/brown spot	–	–
L8	Tambov region (Alouette)	Early blight/brown spot	Aalt	Aalt (32.5)
S1	Moscow region, Russia (Red Scarlett)	Stem canker (*R. solani*)	Rsol	Rsol
S2	Moscow region, Russia (Red Scarlett)	Black dot (*C. coccodes*)	Ccoc	Ccoc (32.1)
S3	Tyumen region, Russia (Red Scarlett)	Stem canker	Rsol	Rsol (30.1)
S4	Tyumen region, Russia (Evolution)	Stem canker	Rsol	Rsol (26.1)
S5	Tyumen region, Russia (Red Scarlett)	Black dot suspected	–	–
S6	Sverdlovsk region, Russia (Rozara)	Stem canker, black dot	Rsol, Ccoc,	Rsol (32.0) Ccoc (30.0)
S7	Sverdlovsk region, Russia (Rozara)	Stem canker, black dot (suspected)	Rsol Ccoc	Rsol (33.2) Ccoc (34.0)
S8	Sverdlovsk region, Russia (Gala)	Stem canker	Rsol	Rsol (24.3)
S9	Kurgan region, Russia (Rozara)	Stem canker	Rsol	Rsol (34.0)
S10	Chelyabinsk region, Russia (Red Scarlett)	Stem canker	Rsol	Rsol (34.2)
S11	Chelyabinsk region, Russia (Red Scarlett)	Black dot	Ccoc	Ccoc (35.0)
S12	Chelyabinsk region, Russia (Red Scarlett)	Stem canker	Rsol	Rsol (32.7)
S13	Chelyabinsk region, Russia (Red Scarlett)	Stem canker suspected	–	–

^1^ T, L, and S indicate potato tubers, leaves, and stems, respectively. ^2^ Potato cultivar is indicated in brackets (if known). ^3^ Pathogen designations: Asol, *Alternaria solani*; Aalt, *A. alternata*; Rsol, *Rhizoctonia solani*; Ccoc, *Colletotrichum coccodes*; Sss, *Spongospora subterranea*; Fus, *Fusarium* spp. ^4^ C_t_ values are shown in brackets. ^5^ The sample was collected from a tomato plant.

## Supplementary Materials

The following are available online at http://www.mdpi.com/2079-6374/8/4/129/s1, **Figure S1.** Alignment of DNA sequences in the regions used for primer design for *Alternaria solani*, *Alternaria alternata*, and *Rhizoctonia solani*; **Figure S2.** Alignment of DNA sequences in the regions used for primer design for *Colletotrichum coccodes* and *Spongospora subterranea*; **Figure S3.** Alignment of DNA sequences in the regions used for genus-specific primer design for *Fusarium* spp.; **Figure S4.** Examples of fluorescent curves illustrating specificity of the test systems developed for detection and identification of *Alternaria solani*, *A. alternata*, *Rhizoctonia solani*, *Colletotrichum coccodes*, *S. subterranea*, and *Fusarium* spp.; **Figure S5.** Standard regression curves of a serial dilution of DNA from the target potato pathogens obtained for freeze-dried microarrays. **Table S1.** Effect of annealing temperature on the efficiency of test systems developed for detection of target fungal pathogens of potato.
