# Supplementary material for: Preserved Microarrays for Simultaneous Detection and Identification of Six Fungal Potato Pathogens with the Use of Real-Time PCR in Matrix Format
Source: Biosensors (Basel). 2018 Dec 13;8(4):129. doi: 10.3390/bios8040129 (PMC6316111; doi:10.3390/bios8040129)
Supplement: Supplementary file 1 [file biosensors-08-00129-s001.pdf]

Supplementary Materials

Article

Preserved Microarrays for Simultaneous Detection and Identification of Six Fungal Potato Pathogens with the Use of Real-Time PCR in Matrix Format

Maksim Nikitin [1], Ksenia Deych [1], Inessa Grevtseva [1], Natalya Girsova [2], Maria Kuznetsova [2], Mikhail Pridannikov [2,3], Vitaly Dzhavakhiya [2], Natalia Statsyuk [2,*] and Alexander Golikov [1]

[1] GenBit LLC, Nauchny pr., 20, Bld. 4, Moscow 117246, Russia; nikitin@genbitgroup.com (M.N.); ksedeych@gmail.com (K.D.); grevtseva@genbitgroup.com (I.G.); golikov@genbitgroup.com (A.G.)
[2] All-Russian Research Institute of Phytopathology, Institute Str., 5, Bolshie Vyazemy 143050, Russia; ngirsova@yandex.ru (N.G.); kuznetsova@vniif.ru (M.K.); mikhail.pridannikov@yahoo.com (M.P.); dzhavakhiya@yahoo.com (V.D.)
[3] Centre of Parasitology, Severtsov Institute of Ecology and Evolution, Russian Academy of Sciences, Leninskii Prospect 33, 119071, Moscow, Russia
[*] Correspondence: nataafg@gmail.com; Tel.: +7-916-304-6235

(**a**)

```
A. solani_HE796714.1          GAAGGTCAGCGACGAGTAAGTTGCCCTCACGCTTTTCACCACCTTTTACATTCAGATATACTAACGCTT-TTCCAGCATCA
A. infectoria_KJ920982.1      GGGTGTCAGCGAAGAGTAAGTCCCATA----CCTTTGAATGCGCGGTCCGTTCAAATTTGCTAACATTT-CCTCAGCCTCA
A. ethzedia_AY563284.1        GGGTGTCAGCGACGAGTAAGTTTTTCCCATGCCTTTGAATGCGCGGTCCGTTCAAATTTGCTAACATTT-CCTCAGCCTCA
A. radicina_AY563286.1        GAACGTCAGCAACGAGTAAGTCTTATCCAAACCTTGAAAAATCCACATTTACAGATATACTAACATTTCTCTTTAGCACCA
A. chartarum_AY563319.1       GAAGGTCAGCGACGAGTAAGTTTTCTCCAGACTCTAGAACACTTCGCATTTTCAGATATGCTAACATTTTCCTCAGCATCA
A. arborescens_KY626430.1     GAAGGTTAGCGACGAGTAAGTTATCCTTGTACCTTCGATTACTTCGCAGATTCAGATATACTAACATAT-CCCCAGCATCA
A. tenuissima_KP341692.1      GAAGGTTAGCGACGAGTAAGTTACCCTTGTACCTTCGATTACTTCGCAGATTCAGATATACTAACATAT-TCCCAGCATCA
A. alternate_JQ282273.1       GAAGGTTAGCGACGAGTAAGTTACCCTTGTACCTTCGATTACTTCGCAGATTCAGATATACTAACATAT-TCCCAGCATCA
A. longipes_JQ004405.1        GAAGGTTAGCGACGAGTAAGTTACCCTTGTACCTTCGATTACTTCGCAGATTCAGATATACTAACATGT-TTCCAGCATCA
A. scirpicola_AY563320.1      GGAAGTTAGCGACGAGTAAGTTTTTGCCATGTTCTCAGACACTGCAACTGTTCAGATATGCTAATATATTTCACAGCGTCA
                              *   ** ** ** *    ***   ** *** * ***                          *           ***  **
```

(**b**)

```
A.alternata_FN689397.1        AGTTACCCTTGTACCTTCGATTACTTCGCAGATTCAGATAT--ACTAACATATTCCCAGCATCACCTATGTCGCTACCGCCACTCTTCCCAACTACTGC
A.tenuissima_KJ921019.1       AGTTACCCTTGTACCTTCGATTACTTCGCAGATTCAGATAT--ACTAACATATTCCCAGCATCACCTATGTCGCTACCGCCACTCTTCCCAACTACTGC
A.arborescens_KY626430.1      AGTTATCCTTGTACCTTCGATTACTTCGCAGATTCAGATAT--ACTAACATATCCCCAGCATCACCTATGTCGCTACCGCCACTCTTCCCAACTACTGC
A.longipes_KP123990.1         AGTTACCCTTGTACCTTCGATTACTTCGCAGATTCAGATAT--ACTAACACATTCCCAGCATCACCTACGTCGCTACCGCCACTCTTCCCAACTACTGC
A.solani_HE796710.1           AGTTGCCCTCACGCTTTTCACCACCTTTTACATTCAGATAT--ACTAACGCTTTTCCAGCATCACCTACGTCGCTACCACCACTCTTCCCAACTACTGC
A.radicina_EU139341.1         AGTCTTATCCAAACCTTGAAAAAT-CCACATTTACAGATATACTAACATTTCTCTTTAGCACCACCTATGTCGCCACTACAACCATTCCTAGCTACTGC
A.infectoria_KJ920982.1       AGTCCCATACCTTTGAATGCG----CGGTCCGTTCAAA--TTTGCTAACATTTCCTCAGCCTCACCTATGTCGGTACCACCACTCTTCCCAGCTACTGC
                              ***                                *    *           *    ***  *****  ****  **  * **  **** * *******
```

(**c**)

```
R. solani KX129968.1          CTTCACACCTGCTCCTCTTT-GTGTATTAGCTGGATCTCAGTGTTATGCTTGGTTCCACTCAGCGTGA
R. cerealis AF222793.1        ATTCACGTCTGCTCCTCTTAAATGCATTAGCTGGATCTCTATAA---AACCGGTTCCACTCGGCGTGA
R. oryzae KX468799.1          --GCAAGTCGACTCTTCTTAAATGCATTAGCTGGGGTTTATATAGTTGGATCCTTGGTGTGATAATTA
R. crocorum AB044000.1        ---CTAGTCAGCTTGCTTTAAATACATCAGCCGGCTCTGTACTT---GATCGGTCCGACTCGACGTGA
                                      *             *   *  *  *                    *     *     * *

R. solani KX129968.1          TAAATTATCTAT---CGCTGAGGACACTGTAAAAAGTGGCCAAGG-TAAATGCAGATGAACCGCTTCT
R. cerealis AF222793.1        TAA-GTATCACT---CGCTGAGGACACTCTTGCAAAAGGGTGGCCGGATTCATGGACGAACCGCTTCT
R. oryzae KX468799.1          TCTACGCCTTGAAGTCCCTGTAGACTCTGCTTCAAATCGT----------------------------
R. crocorum AB044000.1        TAACATCTTCGTTGAGGATT---GCATTGACATATAAATGCAGCCGGAAAGTAC--------------
                                                *              *
```

Figure S1. Alignment of DNA sequences in the regions used for primer design for (**a**) *Alternaria solani*, (**b**) *Alternaria alternata*, and (**c**) *Rhizoctonoa solani*. To illustrate primer specificity, sequences of each target pathogen (indicated by red color) were aligned with sequences of related species of the same genus. Identical nucleotides are indicated with an asterisk and gaps are indicated by dashes. Forward and reverse primers are indicated with yellow and green, respectively; internal probe is indicated with cyan.

(a)

```
C.coccodes KU821179.1          CTAACTTGTTCGAATAGGGTAACCAGATTGGTGCTGCCTTTTGGTGTGTAACCAGACAGACGCCAACGACG
C.acutatum KY049984.1          CTAACTTCTTTGAACAGGGTAACCAGATTGGTGCTGCCTTCTGGTGCGTAGCCAACCGCCAACG----ACG
C.tabaci KM105486.1            AACCTTTCTTCGAATAGGGTAACCAGATTGGTGCTGCCTTTTGGTGCGTAGCCAGACCGACGCCAACGGCT
C.truncatum DQ195751.1         ---------------------------TCGGTGCTGCTTTCTGGTGCGTATCCCAAACGCCGAAGACTCG-
C.gloeosporioides U14138.1     CTAACCTCCTTGTACAGGGTAACCAGATTGGTGCTGCCTTCTGGTACGTGACGAGACCGCCGACGACCCG-
C.asianum JX010406.1           CTAACCTCCTTGTACAGGGTAACCAGATTGGTGCTGCCTTCTGGTACGTGACGAGACCGCCGACGACCCG-
                                                          * ******** ** ****   **   *

C.coccodes KU821179.1          CGGCGATGTTGATCTCCTGAAGAAGATGAATTACTGACTGTGCCCTAGGCAAAACATCTCTGGCGAGCACG
C.acutatum KY049984.1          CGGCGATCTCGATATTTGA-CACGATCTCGTACTGATCTTGGGTACAGGCAAAACATCTCTGGCGAGCACG
C.tabaci KM105486.1            CGACGATATCGACCTCGAA-AGGAAACAACTCATTGACTGTGGCGTAGGCAAAACATCTCTGGCGAGCACG
C.truncatum DQ195751.1         --GTTATATCAACCTC-CAAAGAGTCATGAAGATTAACAATCGAACAGGCAGAACATCTCTGGCGAGCATG
C.gloeosporioides U14138.1     --GCAATAT-ATACTT-GCGAGGACGGCAGATGTTGACGATAGAGTAGGCAAAACATTTCTGGCGAGCACG
C.asianum JX010406.1           --GCAATATCATACTT-GCGAGGACGGCAGATGTTGACGATAGAGTAGGCAAAACATTTCTGGCGAGCACG
                                                                    *        ***** *  ** ***** ***** *
```

(b)

```
S. subterranea AF104308.1          CCTTGAAAGCATGCCTCTTTGAGTGTCGGTTTCTATTCTCCCGGAAACGCCCTGTGCGTGGAAGGGGACTATG
Plasmodiophora brassicae EF195335.1  CCTTGGAAGCATGCCTCTTTGAGTGTCGGTTTCTGTTCGTTTGCGCGCCCGCGCGCGCAAGACAATGAGCTTT
Phagomyxa bellerocheae AF310906.1   ATGCT-TG---------TTTGAGTGTCGTTTCCAATCTAAATTGAACTTTGCCTTGGCATGGTTTTAAGAAAC
Phagomyxa odontellae AF310905.1     ATGCT-TG---------TTTGAGTGTCGTTTCTAATCTAAACTGAACTTTGCTTTGGCATGGTTTTAAGAAAC
Polymyxa graminis EU244488.1       CTCGG-AAGCATGCCTCTCTGAGTATCGGTTTCTATATCTCTCACACTGGTGTGCTGGAGAATGGGAGTTGCT
Ligniera sp. AJ010425.1            CTTCC-GAGCATGCTTCTTTGAGTGTCGGTTTCCATCTCTCACCCCCCCGGGTGAGGACAGTGAGAGTCGGGG
                                                    * ***** *** **    *

S. subterranea AF104308.1          GCTCTGGTCGGTCCATGGCTTGAAAGATTATCCAACCGGTGCGC----------GTCTCTGGCTTCTGATTCG
Plasmodiophora brassicae EF195335.1  G--CTGCGGCATAGCTTGAACGAAGCGACCCGGGATCGTGCGCGCATTGTTGATACCCTCGAGAATGCCGGAG
Phagomyxa bellerocheae AF310906.1   AGAAACTTCTTTGTATT-CG-----------------------------------------------------
Phagomyxa odontellae AF310905.1     AACCCATTTTTTTTGTATTC-----------------------------------------------------
Polymyxa graminis EU244488.1       G--CACCTCTCTGCATCTCCTGAAGTCAACGTCGGCCGA----------------------------AGTCCA
Ligniera sp. AJ010425.1            C--GGTCGCCCTCTCTTGAAATTCGAGGCCGTAAGTCCA----------------------------AGCGAT
                                          *   *
```

Figure S2. Alignment of DNA sequences in the regions used for primer design for (**a**) *Colletotrichum coccodes* and (**b**) *Spongospora subterranea* f.sp. *subterranea*. To illustrate primer specificity, sequences of each target pathogen (indicated by red color) were aligned with sequences of related species of the same and close genera. Identical nucleotides are indicated with an asterisk and gaps are indicated by dashes. Forward and reverse primers are indicated with yellow and green, respectively; internal probe is indicated with cyan.

F. redolens HM057339.1	GACAGTATTTCACAGACCGGTCACTTGATCTACCAGTGCGGTGGTATCGACAAGCGAA…
F. sacchari JF740818.1	GACATGGCTACACAGACCGGTCACTTGATCTACCAGTGCGGTGGTATCGACAAGCGAA…
F. avenaceum JX978485.1	-------CTTGACAGACCGGTCACTTGATCTACCAGTGCGGTGGTATCGACAAGCGAA…
F. foetens JF740825.1	GACATCGTTTCACAGACCGGTCACTTGATCTACCAGTGCGGTGGTATCGACAAGCGAA…
F. oxysporum JF740878.1	GACATCGTTTCACAGACCGGTCACTTGATCTACCAGTGCGGTGGTATCGACAAGCGAA…
F. proliferatum FJ538244.1	GACATCGCTTCACAGACCGGTCACTTGATCTACCAGTGCGGTGGTATCGACAAGCGAA…
F. solani LC224295.1	GACAATCATCTACAGACCGGTCACTTGATCTACCAGTGCGGTGGTATCGACAAGCGAA…
F. culmorum JF740860.1	GACATACTTTGATAGACCGGTCACTTGATCTACCAGTGCGGTGGTATCGACAAGCGAA…
F. graminearum JF740871.1	GACATACTTTGATAGACCGGTCACTTGATCTACCAGTGCGGTGGTATCGACAAGCGAA…
F. sambucinum KC899120.1	-GACATACATCATAGACCGGTCACTTGATCTACCAGTGCGGTGGTATCGACAAGCGAA…

F. redolens HM057339.1	…TCAAGTACGCCTGGGTTCTTGACAAGCTCAAAGCCGAGCGTGAGCGTGGTATCACCAT
F. sacchari JF740818.1	…TCAAGTACGCCTGGGTTCTTGACAAGCTCAAGGCCGAGCGTGAGCGTGGTATCACCAT
F. avenaceum JX978485.1	…TCAAGTACGCCTGGGTTCTTGACAAGCTCAAAGCCGAGCGTGAGCGTGGTATCACCAT
F. foetens JF740825.1	…TCAAGTACGCCTGGGTTCTTGACAAGCTCAAGGCCGAGCGTGAGCGTGGTATCACCAT
F. oxysporum JF740878.1	…TCAAGTACGCCTGGGTTCTTGACAAGCTCAAGGCCGAGCGTGAGCGTGGTATCACCAT
F. proliferatum FJ538244.1	…TCAAGTACGCCTGGGTTCTTGACAAGCTCAAGGCCGAGCGTGAGCGTGGTATCACCAT
F. solani LC224295.1	…TCAAGTACGCCTGGGTCCTTGACAAGCTCAAGGCCGAGCGTGAGCGTGGTATCACCAT
F. culmorum JF740860.1	…TCAAGTACGCCTGGGTTCTTGACAAGCTCAAAGCCGAGCGTGAGCGTGGTATCACCAT
F. graminearum JF740871.1	…TCAAGTACGCCTGGGTTCTTGACAAGCTCAAAGCCGAGCGTGAGCGTGGTATCACCAT
F. sambucinum KC899120.1	…TCAAGTACGCTTGGGTTCTTGACAAGCTCAAAGCCGAGCGTGAGCGTGGTATCACCAT

Figure S3. Alignment of DNA sequences in the regions used for genus-specific primer design for *Fusarium* spp. Gaps are indicated by dashes; forward and reverse primers are indicated with yellow and green, respectively; internal probe is indicated with cyan.

Table S1. Effect of annealing temperature on the efficiency of test systems developed for detection of target fungal pathogens of potato

Annealing temperature (T_a), °C	Maximum fluorescence level (F_m)	Threshold fluorescence level (F_t)	Threshold cycle (C_t)
**Asol4** (*Alternaria solani*)			
56	300	95.4	39.14
58	6900	120.6	29.44
60	7500	113.4	27.23
62	200	44.3	43.15
**Aalt1** (*Alternaria alternata*)			
56	5700	200.4	17.21
58	9200	189.3	18.34
60	10000	215.5	16.20
62	13000	243.0	20.60
**Rsol4** (*Rhizoctonia solani*)			
56	4200	152.1	21.69
58	7100	146.9	22.87
60	7500	133.2	20.24
62	8500	144.3	23.25
**Ccoc** (*Colletotrichum coccodes*)			
56	3000	112.3	29.48
58	3500	108.4	27.67
60	4200	104.3	25.89
62	1700	85.3	36.59
**Sss** (*Spongospora subterranea*)			
56	1900	95.6	18.59
58	5600	104.7	18.21
60	7600	114.1	18.30
62	720	88.4	22.28
**Fus** (*Fusarium* spp.)			
56	1700	55.3	18.42
58	1800	48.2	17.58
60	1700	66.1	13.82
62	590	38.2	29.83

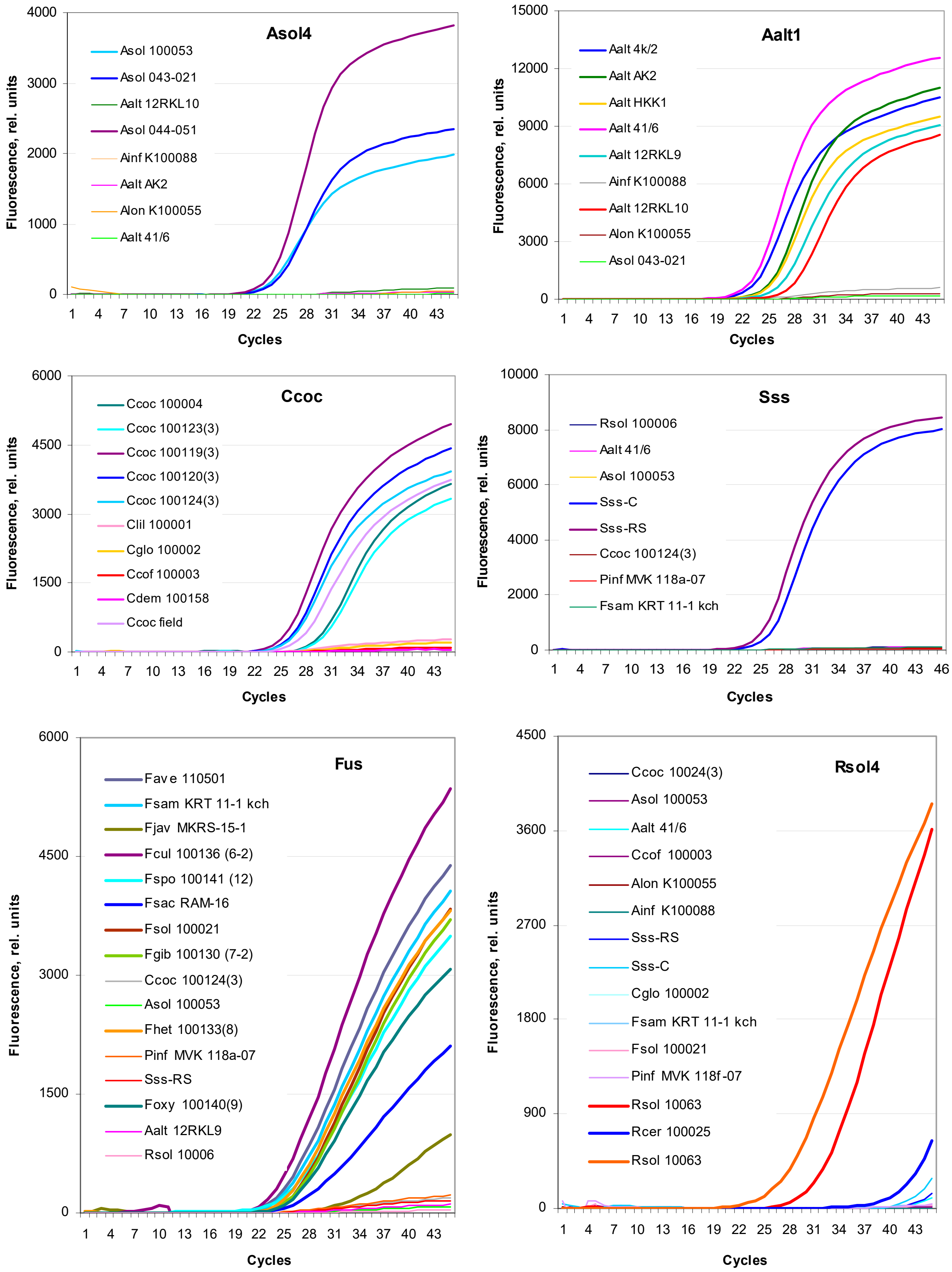

Figure S4. Examples of fluorescent curves illustrating specificity of the test systems developed for detection and identification of *Alternaria solani* (Asol4), *A. alternata* (Aalt1), *Rhizoctonia solani* (Rsol4), *Colletotrichum coccodes* (Ccoc), *S. subterranea* (Sss), and *Fusarium* spp. (Fus). Curves for target species indicated with bold lines. Indications: Asol, *A. solani*; Aalt, *A. alternata*; Ainf, *A. infectoria*; Alon, *A. longipes*; Ccoc, *C. coccodes*; Clil, *C. lilii*; Cglo, *C. gloeosporioides*; Ccof, *C. coffeanum*; Cdem, *C. dematium*; Sss, *S. subterranea*; Rsol, *R. solani*; Rcer, *R. cerealis*; Fave, *F. avenaceum*; Fsam, *F. sambucinum*; Fjav, *F. javanicum*; Fcul, *F. culmorum*; Fsp, *F. sporotrichioides*; Fsac, *F. sacchari*; Fsol, *F. solani*; Fgib, *F. gibbosum*; Fhet, *F. heterosporum*; Foxy, *F. oxysporum*; Pinf, *Phytophthora infestans*.

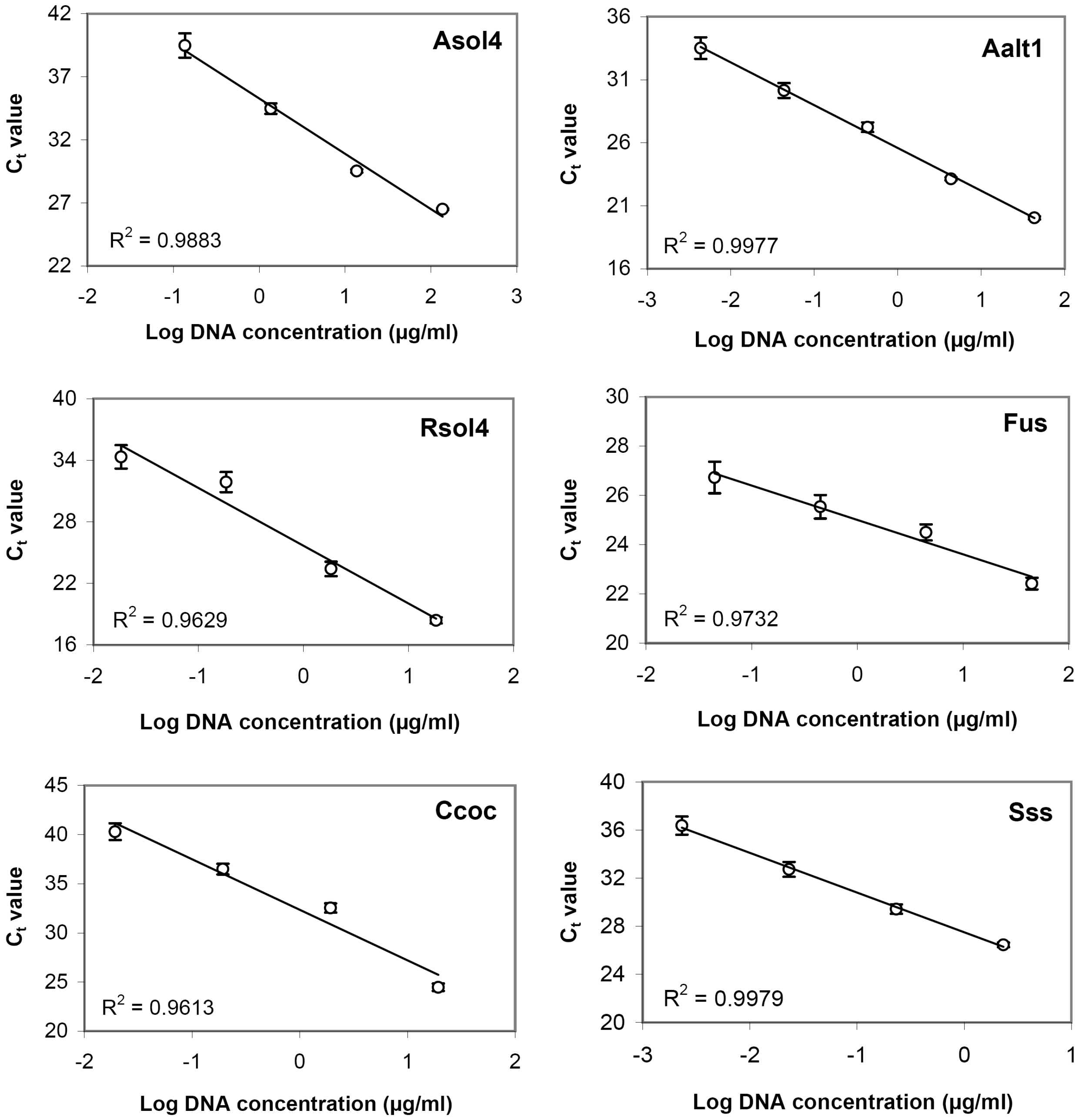

Figure S5. Standard regression curves of a serial dilution of DNA from the target potato pathogens obtained for freeze-dried microarrays. Test systems designations: **Asol4**, *Alternaria solani;* **Aalt1**, *A. alternata;* **Rsol4**, *Rhizoctonia solani;* **Ccoc**, *Colletotrichum coccodes;* **Sss**, *Spongospora subterranea;* **Fus**, *Fusarium* spp.
